# U1 snRNP and RNA polymerase II interaction is predominantly mediated by Prp40 rather than U1-70K in yeast

**DOI:** 10.1093/nar/gkag581

**Published:** 2026-06-10

**Authors:** Xueni Li, Jiaqin Li, Shasha Shi, Zhiling Kuang, Pankaj Srivastava, Aaron Issaian, Shaun Bevers, Marisa Elise Wagley, Angelo D’Alessandro, Rui Zhao

**Affiliations:** Department of Biochemistry and Molecular Genetics, University of Colorado Anschutz Medical Campus, Aurora, CO 80045,United States; Department of Biochemistry and Molecular Genetics, University of Colorado Anschutz Medical Campus, Aurora, CO 80045,United States; Department of Biochemistry and Molecular Genetics, University of Colorado Anschutz Medical Campus, Aurora, CO 80045,United States; Department of Biochemistry and Molecular Genetics, University of Colorado Anschutz Medical Campus, Aurora, CO 80045,United States; Department of Biochemistry and Molecular Genetics, University of Colorado Anschutz Medical Campus, Aurora, CO 80045,United States; Department of Biochemistry and Molecular Genetics, University of Colorado Anschutz Medical Campus, Aurora, CO 80045,United States; Department of Biochemistry and Molecular Genetics, University of Colorado Anschutz Medical Campus, Aurora, CO 80045,United States; Department of Biochemistry and Molecular Genetics, University of Colorado Anschutz Medical Campus, Aurora, CO 80045,United States; Department of Biochemistry and Molecular Genetics, University of Colorado Anschutz Medical Campus, Aurora, CO 80045,United States; Department of Biochemistry and Molecular Genetics, University of Colorado Anschutz Medical Campus, Aurora, CO 80045,United States

## Abstract

Transcription and splicing are coupled both temporally and physically. A previous cryo-EM structure of the human U1 snRNP and RNA polymerase pol II complex has shown that U1 snRNP uses predominantly the RRM domain of U1-70K to directly interact with the RPB2 subunit of pol II. However, residues on U1-70K involved in the interaction with pol II are not conserved in yeast U1-70K, raising the question whether yeast U1 snRNP interacts with pol II in a similar manner. We found that yeast pol II directly interacts with U1 snRNPs, but U1-70K makes a minimal contribution to this interaction. On the other hand, multiple domains of yeast Prp40 interact with pol II and the removal of the C-terminal domain (CTD) of pol II does not affect this interaction. Although yeast Prp40 is stably associated with U1 snRNP, its human homologs, PRPF40a and PRPF40b, are alternative splicing factors that are not integral components of U1 snRNP. The lack of a stable association between human PRPF40s and U1 snRNP, together with the exposed RRM domain of U1-70K in the absence of PRPF40s, allows U1-70K to serve as the primary mediator of the interaction between U1 snRNP and pol II in humans.

## Graphical Abstract

**Graphical Abstract:**
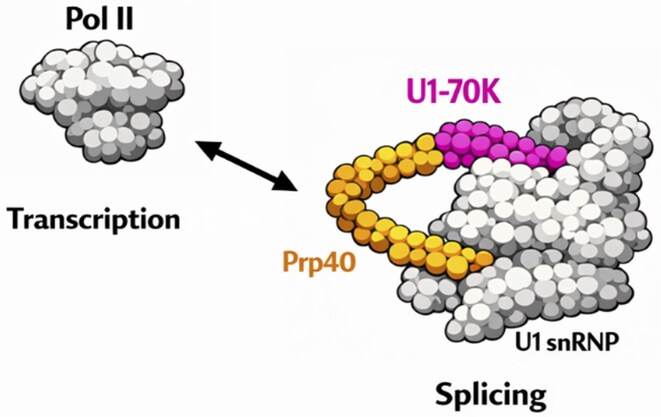

## Introduction

In eukaryotes, DNA is first transcribed into pre-mRNA, which undergoes multiple processing events including splicing, before becoming mature messenger RNA (mRNA). The two critical events in this process, transcription and splicing, are catalyzed by RNA polymerase II (pol II) and the spliceosome, respectively. Pol II, the main eukaryotic transcription machinery for protein-coding genes, contains 12 subunits, RPB1-12. The largest subunit, RPB1, contains a C-terminal domain (CTD) with multiple heptapeptide (YSPTSPS) repeats, 52 in human and 26 in yeast, that are heavily regulated by phosphorylation [1]. The splicing machinery, the spliceosome, contains 5 snRNPs (U1, U2, U4, U5, and U6) and numerous non-snRNP factors [2]. Spliceosome components assemble on pre-mRNA in a stepwise manner forming at least 10 distinct complexes. U1 snRNP, SF1 (BBP in yeast), and the U2AF35/65 heterodimer first recognize the 5′ ss, BPS, and 3′ ss, respectively, forming the E complex. Subsequently, the U2 snRNP replaces SF1 to first form a pre-A complex [3], followed by formation of the A complex. Next, the U4/U6.U5 tri-snRNP joins the spliceosome to form the Pre-B complex, which undergoes further rearrangement, forming the B, B^act^, B*, C, C*, P, and ILS complex sequentially to remove the intron and ligate the exons.

Splicing and transcription are coupled both temporally and spatially. For example, a high percentage of splicing occurs co-transcriptionally and the speed of transcription can regulate splicing [4]. The transcription and splicing machinery also interact physically. U1 snRNP is responsible for the initial recognition of the 5′ splice site (ss) and plays a key role in mediating the coupling between splicing and transcription. U1 snRNP has been observed to associate with pol II [5, 6], although it was unclear for a long time whether this interaction is direct. In 2021, Dr. Patrick Cramer’s lab determined a cryo-EM structure of human pol II and U1 snRNP assembled on a DNA–RNA scaffold made of a DNA mismatch bubble and a pre-mRNA containing the 5′ ss [7]. This structure reveals that the RRM domain of U1-70K directly interacts with the RPB2 subunit of pol II. However, none of the human U1-70K residues that interact with RPB2 are conserved in yeast, making it unclear whether yeast U1 snRNP also interacts with pol II in a similar manner.

Yeast U1 snRNP is much larger and more complex than human U1 snRNP [8]. The human U1 snRNP is composed of a 164-nt U1 small nuclear RNA (snRNA), seven Sm proteins, U1A, U1C, and U1-70K proteins. The yeast U1 snRNP contains a much larger U1 snRNA (568 nt) and homologs of all the human U1 snRNP proteins (referred to as the U1 snRNP core). The yeast U1 snRNP core resembles the entire human U1 snRNP, with seven additional stably associated auxiliary proteins (Prp40, Luc7, Snu71, Prp39, Prp42, Snu56, and Nam8) [8]. It has been previously reported that yeast Prp40 interacts with pol II [5]. It remains unclear whether U1-70K and Prp40 both interact directly with pol II and what their relative contributions are to the U1 snRNP–pol II interaction. In this paper, we set out to answer these questions using mostly biochemical approaches.

## Materials and Methods

### Human pol II and U1-70K purification and pull-down experiments

Truncated human U1-70K (residue 1–216) with a C-terminal 6xHis tag was cloned into pET28. A triple mutation (K118E/R121E/E124K) and a ΔRRM variant (residues 102–181 deleted) were generated on this construct using NEBuilder (NEB). The plasmids were transformed into BL21 (DE3), and proteins were expressed at 16 °C overnight in 2xYT media. Cells were lysed by sonication in lysis buffer containing 20 mM Hepes pH 7.5, 1 M NaCl, 1 M Urea, and 5 mM TCEP. Proteins were purified using Ni-NTA resin (Invitrogen, catalog # R90101) and eluted in lysis buffer with a discontinuous imidazole gradient from 50 to 300 mM, increasing by 50 mM every 5 ml. The eluted proteins were dialyzed against lysis buffer to remove imidazole and concentrated to ∼5 mg/ml.

Human pol II was purified from nuclear extract [9] (prepared from HEK293 free style suspension cells harvested at a density of ∼2 million/ml) using 8WG16 antibody bound to protein A/G resin. The resin was washed using 5 × 20 bed volume (BV) of washing buffer containing 20 mM Hepes pH 7.5, 800 mM NaCl, 1 mM TCEP, and 0.02% NP-40, followed by additional washing steps as described below: 1 × 20BV of 20 mM Hepes pH 7.5, 600 mM NaCl, 1 mM TCEP, 0.02% NP-40; 1 × 20BV of 20 mM Hepes pH 7.5, 450 mM NaCl, 1 mM TCEP, 0.02% NP-40; 1 × 20BV of 20 mM Hepes 7.5, 300 mM NaCl, 1 mM TCEP, 0.02% NP-40; 2 × 20BV of 20 mM Hepes pH 7.5, 150 mM NaCl, and 1 mM TCEP. Purified pol II on protein A/G resin (UBPbio, catalog #P5030-5) was incubated with 2.5 μM purified U1-70K protein in SEC150 buffer containing 20 mM Hepes pH 7.5, 150 mM NaCl, 3 mM MgCl_2_, 1 mM TCEP, at 4°C for 1 h. Resins were washed four times with SEC150 buffer, and the bound proteins were loaded onto SDS-PAGE for subsequent Western blot analysis using an anti-His antibody (UBPbio, catalog #Y1011). Mouse IgG (Diagenode, catalog # C15400001), used in place of the pol II antibody 8WG16 and bound to protein A/G resin, was subjected to the same pol II purification procedures and served as the IgG control.

### Yeast pol II pull-down and snRNA detection

RPB3 TAP tagged yeast strains expressing RPB1 wild-type, CTD 11 2/7 and 10 5/7 truncations were gifts from Richard Young’s lab [10] . 200 ml yeast cells were cultured to an OD600 of 3 at 30 °C, harvested and lysed using bead-beating methods in two times of wet cell volume of buffer IgG 150 (20 mM Tris-HCl pH 8.0, 150 mM NaCl, 0.02% NP-40, and 1 mM dithiothreitol (DTT) supplemented with protease inhibitors (Sigma–Aldrich, catalog # S8830). The lysate was first cleared by a centrifugation at top speed in a microfuge for 15 min, then an ultracentrifugation in Ti70.1 at 42 000 rpm for 1 h, at 4 °C. The cleared cell lysate was incubated with 30 μl of IgG resin for 3 h to pull down pol II. The resins were washed with 7 × 1 ml IgG 150 buffer, and pol II was cleaved from the resin in 20 μl of IgG 150 buffer without NP-40, containing 1.5 μg of TEV protease, overnight at 4 °C. RNAs in 5–10 μl elutions or 1–2 μl of lysate were released using proteinase K digestion and analyzed by solution hybridization with IRDye700-labeled probes specific for U1, U2, U4, U5, and U6 snRNAs [11].

### Generating U1-70K mutant yeast strains

To assess the effects of mutations on the RRM domain of yeast U1-70K (SNP1) mutations, we integrated a TAP tag to the C termini of RPB3 using *HIS3* marker in the *snp1Δ* [pRS316-SNP1] shuffle strain (a gift from Stewart Shuman’s lab) [12]. A 1.59-k bp DNA segment bearing the SNP1 gene (nucleotides −400 to +1190) was amplified from pRS413-SNP1 (a gift from Stewart Shuman’s lab) [12] and inserted into pRS415 vector to make pRS415-SNP1. E122K, K125E, K129E, F130A, and K156E single mutations or Δ108–188, Δ93–247 deletions were generated on pRS415-SNP1. The plasmids were transfected into the RPB3-TAP SNP1 shuffle strain. After FOA selection, the resulting strains were used to perform the same pol II pulldown and snRNA detection experiment as above.

### Pol II purification

Yeast pol II was purified by using the TAP tag on RPB3 [10]. Specifically, 6 L of cells were cultured in YEPD medium at 30 °C to an OD600 of 4. The cell pellets were re-suspended in 10 ml of lysis buffer (50 mM Tris–HCl, pH 8.0, 200 mM NaCl, 0.05% NP-40, and 1 mM DTT). The cell suspension was snap-frozen in liquid nitrogen to form yeast “popcorn” and cryogenically grounded using a SPEX 6870 Freezer/Mill. The frozen cell powder was thawed at room temperature and re-suspended in an additional 60 ml of lysis buffer with protease inhibitor cocktails (Sigma–Aldrich, catlog # P8849). The cell lysate was first centrifuged at 27 845 × *g* for 1 h in a GSA rotor (Sorvall), and the supernatant was further centrifuged at 167 424 × *g* in a 45Ti rotor (Beckman) for 1 h at 4 °C. The supernatant was incubated with 2 ml of IgG Sepharose-6 Fast Flow resin (GE Healthcare, catalog # 17096901) overnight at 4°C. The resin was washed with IgG washing buffer (20 mM Tris–HCl pH 8.0, 200 mM NaCl, 0.02% NP40, and 0.5 mM DTT) and then washed with the same buffer containing 500 mM NaCl then 750 mM NaCl. The resin was incubated with TEV protease in 1 ml TEV150 buffer (20 mM Tris–HCl pH 8.0, 150 mM NaCl, and 0.5 mM DTT) overnight at 4 °C. The purified pol II was slightly concentrated using an Amicon centrifugal filter (Millipore, catalog # UFC8100), aliquoted and frozen at -80 °C until use.

To purify pol II with the entire CTD truncated, we inserted a PreScission cutting site into RPB1 before its CTD region (between residues 1513 and 1514) and the modified plasmid was transformed into RPB3 TAP-tagged yeast strains for the RPB1 shuffling experiment (strain GHY1729 [10]). After FOA selection, the strain was cultured and the variant pol II was purified in the same way described above, except for the addition of an incubation step of the resin with PreScission protease and then washes to remove the CTD region, before the final release of purified pol II using TEV protease.

### U1 snRNP purification

To purify wild-type and SNP1-RRM deleted U1 snRNP, we integrated a STP tag (SBP-TEV-protA) to the C terminus of U1A using *HIS3* marker in *snp1Δ* [pRS316-SNP1] strain, transformed pRS415-SNP1-2xHA, pRS415-SNP1(Δ108–188)-2xHA into it and shuffle out pRS316-SNP1 using FOA selection. Twelve liters of cells of each SNP1 construct were cultured in YEPD medium at 30 °C to an OD600 of 4 and harvested for U1 snRNP purification.

To purify PRP40-depleted U1 snRNP, we introduced an STP tag (SBP-TEV-protA) to the C-terminus of U1A using *HIS3* marker in *Luc7Δ* [pRS316-LUC7] strain [13]. The plasmid bearing auxin-inducible degron (AID) plus 3x-HA tag at the C terminus of LUC7 and an auxin receptor (OsTIR1) driven by a β-estradiol inducible promoter [14] was transformed into the U1A STP-tagged *luc7* [pRS316-LUC7] strain. After FOA selection, the strain was cultured in YEPD medium at 30 °C to an OD600 of 1, and 1 ml each of β-estradiol (0.116 g/50 ml of ethanol) and IAA (6.58 g/50 ml of ethanol) were added per liter cell to induce the degradation of LUC7 for 4 hr. Twelve liters of cells were cultured and harvested for U1 snRNP purification. U1 snRNP purification was performed essentially the same way as previously described [8], but without the second purification using calmodulin resin (GEHealthcare, catalog # 17052901).

### Pull-down assay to evaluate the interaction between pol II and U1 snRNP *in vitro*

Purified pol II and U1 snRNP were incubated at ∼1:1 molar ratio in a buffer containing 20 mM Tris–HCl pH 8.0, 150 mM NaCl, 2 mM MgCl_2_, 1 mM CaCl_2_, and 0.5 mM DTT for 2 h at 4°C. Pol II and associated U1 snRNP were pulled down using calmodulin resin, taking advantage of the CBP tag remained on pol II after purification. The resins were washed using the same buffer as above, and proteins were eluted using a buffer containing 20 mM Tris–HCl pH 8.0, 150 mM NaCl, 2 mM EGTA, and 0.5 mM DTT. The eluted proteins were separated on SDS–PAGE and transferred to a nitrocellulose membrane. Western blots were performed to detect RPB1, RPB3-CBP, U1A-SBP, or SNP1-2xHA. Antibodies used were RPB1 antibody (8WG16 [15]), CBP tag antibody (Genscript, catalog # A00635), SBP tag antibody (Santa Cruz, catalog # sc-101595), and HA tag antibody (Roche, clone 3F10, catalog # 11867423001).

### Pull-down assay to evaluate the interaction between pol II and U1-70K ΔN, PRP40, SNU71, and LUC7

The coding regions of yeast PRP40, SNU71, and LUC7 full length and N-terminal truncated SNP1 (SNP1 Δ1–97) were amplified by PCR using genomic *Saccharomyces cerevisiae* DNA as a template and ligated into pRS414, pRS416, and pRS317 vectors. The final plasmids constructed are: pRS414/GPD-protA-TEV-PRP40, pRS414/GDP-protA-TEV-SNU71, pRS414/GPD-protA-LUC7, and pRS414/GPD-protA-TEV-SNP1 Δ1–97. Yeast BCY123 cells transformed with each plasmid were maintained and cultured using the appropriate selective media. One hundred to 500 ml of cells cultured at 30 °C to an OD600 of 3–4 were used for purification. Cells were harvested and lysed in lysis buffer (40 mM Tris–HCl pH 8.0, 200 mM NaCl, 0.05% NP40, 1 mM DTT, protease inhibitors, and 5 μl Benzonase/ml) using the bead-beating method. The lysates were applied to IgG resin. The resins were washed with the lysis buffer and incubated with 0.4 μM purified pol II (TEV protease contains a His tag and was removed from pol II using Ni-NTA resin) in a buffer containing 20 mM Tris–HCl pH 8.0, 150 mM NaCl, and 0.5 mM DTT for 1.5 h at 4 °C. The resins were washed, and the bound proteins were further analyzed on SDS–PAGE and transferred to a nitrocellulose membrane. Western blot was performed using RPB1 antibody and anti-CBP antibody as the above.

### GST pull-down assays using purified proteins

Various PRP40 domains fused with an N-terminal Flag tag and SNP1 RRM domain (residue 98–188) were cloned into the pGEX-6p-1 vector (GE Healthcare) and purified from *Escherichia coli* as GST fusion proteins. The Prp40 domains constructed are: 1–75 (WW); 134–189 (FF1); 204–269 (FF2); 270–335 (FF3); 351–415 (FF4); 425–488 (FF5); 488–552 (FF6); 488–583 (FF6 ext); and 351–552 (FF4-6). To detect interactions between Prp40 domains with pol II, GST fusion proteins or GST alone were bound on glutathione-Sepharose resin (GE Healthcare, catalog #17015601) and incubated with 1 μM of purified pol II for 1.5 h at 4 °C. The resins were washed, and the proteins were cleaved off the glutathione–Sepharose resin using PreScission protease. The proteins were separated on SDS–PAGE and transferred to a nitrocellulose membrane. Western blot was performed using RPB1 antibody and CBP tag antibody as the above.

### Crosslinking and LC-MS analyses

Purified pol II and Prp40 FF4-6 protein were mixed at ∼1:1.5 molar ratio and dialyzed against buffer (20 mM HEPES pH 7.5, 150 mM NaCl, 1 mM MgCl_2_, and 0.5 mM TCEP). Samples were crosslinked with either 1 mM disuccinimidyl sulfoxide (DSSO, Thermo Fisher catalog # A33545) or 2 mM NHS-Diazirine (SDA, AAT Bioquest, catalog #39006) for 1 h at 4 °C. Residual NHS ester was quenched by the addition of 15 mM ammonium bicarbonate for 30 min. SDA-treated sample was subsequently irradiated with UV light (365 nm, UVP) for 15 min at room temperature. Samples were held at a distance of 5 cm from the light source with no obstruction. Crosslinked samples were diluted with 8 M urea to a final concentration of 2 M urea. DTT and IAA were added to a final concentration of 5 and 10 mM, respectively, and incubated at room temperature for 20 min in the dark. Lys-C (FUJIFILM Wako) and Arg-C Ultra (Promega) were added to the samples at a ratio of 1:50 and incubated overnight at 37°C with constant shaking. Peptide samples were acidified with 0.1% formic acid (FA), pooled, and concentrated to 100 µl. Enrichment of crosslinked peptides was performed by using size-exclusion chromatography (SEC) with a Superdex 30 Increase column (10/300, Cytiva) on an ÄKTA Purifier system (Cytiva) system. Briefly, peptides were separated using isocratic flow (0.1% FA, 30% ACN, and 50 mM NaCl) at a flow rate of 0.7 ml/min. Fractions were collected every 30 s and pooled into groups of three and desalted using Spin Tips (Pierce) for subsequent LC-MS/MS analysis.

Crosslinked peptides were then analyzed by nano-UHPLC-MS/MS (nanoElute 2, timsTOF SCP, Bruker). One microliter of sample was directly loaded onto a PepSep column (150 μm i.d.  ×  250 mm, 1.5 μm C18 resin, Bruker). Samples were run at 750 nl/min over a 90 min linear gradient from 4% to 35% ACN with 0.1% FA. The mass spectrometer was operated in positive ion mode. For crosslink peptide identification, the MS1 scan range was 100–1700 *m/z*. The TIMS ramp range was 0.6 to 1.4 1/*k*_0_. The TIMS ramp and accumulation times locked at 75 ms. MS/MS was performed on 5 PASEF ramps per cycle. Data acquisition was performed using Bruker timsControl (version 6.1.1) and Bruker Compass HyStar (version 6.3) software.

Instrument raw files were converted into Mascot Generic Format (MGF) files using ProteoWizard [16]. Converted raw files were loaded into Proteome Discoverer 3.1 and searched against thirteen proteins making up the pol II-Prp40 FF4-6 complex using the MS Annika 3.0 plugin [17]. Search parameters for the search for DSSO crosslinks included carbamidomethylation-C as a fixed modification, oxidation-M, DSSO-STYK, DSSO/amidated-STYK, and DSSO/hydrolysed-STYK as variable modifications, allowing for three missed cleavages. Search parameters for the search for SDA crosslinks included carbamidomethylation-C as a fixed modification, oxidation-M, and SDA-[Any Amino Acid], allowing for three missed cleavages. Precursor mass tolerance was set to 15 ppm, with MS/MS mass tolerance set to 20 ppm. Medium and high confidence FDR cutoffs (peptides and crosslinks) were set to 0.5% and 0.1%, respectively. Results were visualized using xiVIEW [18].

## Results

### The RRM domain of human U1-70K is important for its interaction with pol II

**Figure 1.**
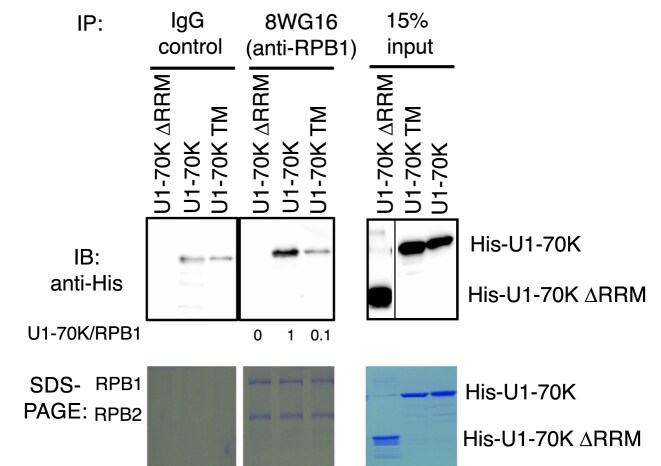
Human U1-70K RRM domain is important for its interaction with pol II. Human pol II purified from HEK293 freestyle cell nuclear extracts using the 8WG16 antibody on protein A/G resin was used to pull down purified truncated U1-70K (residues 1–216), U1-70K (1–216) ΔRRM, and U1-70K (1–216) triple mutant (TM, K118E/R121E/E124K), and probed using an anti-His antibody against the His-tag on U1-70K. Mouse IgG on protein A/G resin was subjected to the same pol II purification procedures and served as the IgG control. The SDS–PAGE below the Western blot demonstrates the quality of the purified pol II (represented by its two largest subunits RPB1 and 2) and U1-70K proteins used in the pull-down assays. IP: immunoprecipitation. IB: Immunoblot. U1-70K/RPB1 ratio is quantified and normalized to WT U1-70K. Shown here is a representative image from five biological replicates.

The cryo-EM structure of human U1 snRNP bound to pol II shows that the RRM domain of U1-70K (particularly residues on helix 1) interacts with pol II [7]. To validate the structural observation, we purified human pol II using the 8WG16 antibody on protein A/G beads. We used purified pol II to pull down purified truncated human U1-70K (residue 1–216, as the full-length U1-70K cannot be expressed and purified from *E. coli*), U1-70K (1–216) ΔRRM, and a U1-70K (1–216) triple mutant of three charged residues on helix 1 (K118E/R121E/E124K), which likely make the most important contribution to pol II interaction based on structural analysis (abbreviated as TM). We showed that both ΔRRM and TM abolished the interaction between U1-70K and pol II (Fig. 1), confirming the importance of RRM domain and these residues in mediating this interaction.

### Yeast pol II preferentially associates with U1 and U2 snRNP

We next set out to investigate the interaction between yeast pol II and U1 snRNP. In the cryo-EM study of human pol II-U1 snRNP complex [7], Zhang *et al*. initially used transcribing pol II, in case active transcription was required for the interaction between pol II and U1 snRNP. However, their structure shows that the interaction is mediated by U1-70K and RPB2 and does not depend on the transcriptional status of pol II. Based on this observation, we designed our experiments using general pol II from yeast whole cell lysate to investigate whether the transcription-independent interaction observed between human U1-70K and pol II is conserved in yeast.

**Figure 2.**
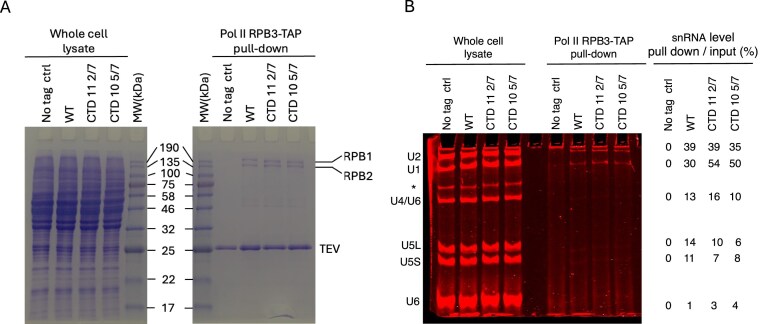
Yeast pol II preferentially associates with U1 and U2 snRNPs. (**A**) pol II WT or CTD truncations (11 2/7 or 10 5/7 heptapeptide repeats remaining) were pulled down from yeast cell lysate on IgG resin using the TAP tag on its RPB3 subunit and visualized on Coomassie-stained SDS–PAGE. (**B**) The pol II pulldown samples were analyzed for its snRNA content using solution hybridization and fluorescently labeled probes for each snRNA. The band above U4/U6, indicated by *, is of unknown origin and does not appear in all solution hybridization gels. snRNA levels in the pull-down samples were quantified and normalized to the input. Shown here is a representative image from three biological replicates.

We pulled down pol II from yeast whole cell lysate carrying the WT or CTD truncated pol II (with 11$\frac{2}{7}$ or 10$\frac{5}{7}$ heptapeptide repeats remaining) using the Tandem Affinity Purification tag composed of CBP-TEV-protein A (TAP) on pol II subunit RPB3 [10] (Fig. 2A). We then detected the snRNAs associated with pol II using solution hybridization [11] with fluorescent probes targeting all 5 snRNAs (U1, U2, U4, U5, and U6). A low level of association was observed between pol II and all snRNAs (Fig. 2B), potentially because some snRNPs and pol II can be tethered to pre-mRNAs. However, the levels of U1 and U2 snRNAs associated with pol II were substantially higher (Fig. 2B), suggesting that yeast pol II preferentially associates with U1 and U2 snRNPs relative to other snRNAs.

### U1-70K mutations and truncations do not substantially reduce pol II and U1 snRNP association in pull-down experiments

**Figure 3.**
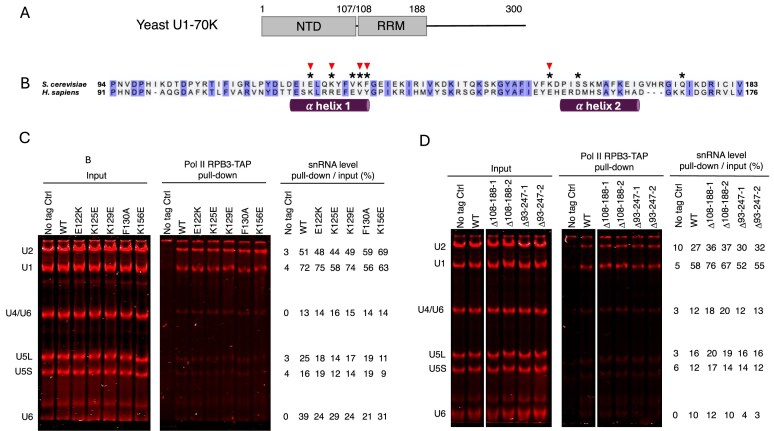
U1-70K mutants and truncations do not substantially reduce pol II and U1 snRNP interaction in pull down experiments from cell lysates. (**A**) Domain organization of yeast U1-70K. (B) Amino acid sequence of the region in human U1-70K RRM domain that interacts with pol II in the cryo-EM structure of U1 snRNP and pol II complex [7] and its sequence alignment with the corresponding region in yeast U1-70K, using the alignment tool in Uniprot. Residues critical for the human U1 snRNP and pol II interaction based on the cryo-EM structure are labeled with *. Residues in U1-70K that were mutated in subsequent experiments are labeled with red triangles. (**C**) pol II was pulled down from yeast strains carrying RPB3-TAP and various single amino acid mutations of yeast U1-70K residues corresponding to critical pol II-interacting residues in human U1-70K. The pull-down was analyzed for its snRNA content using solution hybridization and fluorescently labeled probes for each snRNA. snRNA levels in the pull-down samples were quantified and normalized to the input. Shown here is a representative image from three biological replicates. (**D**) pol II was pulled down from yeast strains carrying RPB3-TAP and U1-70K with two RRM domain truncations (Δ108-188 just removes the RRM domain and Δ93-247 removes a larger region containing the RRM domain which is known to be tolerated by yeast [19] with -1 and -2 representing two biological replicates). The pull-down was analyzed for its snRNA content using solution hybridization and fluorescently labeled probes for each snRNA. snRNA levels in the pull-down samples were quantified and normalized to the input. Shown here are images from two biological replicates.

The cryo-EM structure of the human pol II and U1 snRNP complex showed that U1 snRNP predominantly uses a helix in the RRM domain of U1-70K to interact with RPB2 of pol II [7]. However, the specific amino acids on U1-70K involved in the interaction are not conserved in yeast U1-70K (Fig. 3A and B). To evaluate whether any of the equivalent residues in yeast contribute to pol II and U1 association despite the lack of conservation, we generated five single amino acid mutations on U1-70K (E122K, K125E, K129E, F130A, and K156E) in a U1-70K shuffle strain in which RPB3 of pol II was TAP-tagged. We mutated these residues to the opposite charges, expecting that the mutation would disrupt the U1 and pol II interaction if any of these residues are critical for the interaction. We pulled down pol II using IgG resin through the TAP tag on RPB3 and showed that pol II was associated with similar amounts of U1 and U2 snRNAs from yeast strains expressing the mutant or the WT U1-70K (Fig. 3C).

It is possible that multiple-residue mutations or residues other than the above in the RRM domain of U1-70K interact with pol II. To test this possibility and evaluate the role of the RRM domain of U1-70K in its association with pol II, we generated several U1-70K deletion mutants. Δ93–247 is a deletion containing the RRM domain and surrounding residues that has been shown to be tolerated by yeast [19] and Δ108–188 removes only the RRM domain. We showed that pol II pulled down similar amounts of U1 and U2 snRNAs in yeast carrying these deletions compared to those with the WT U1-70K (Fig. 3D). These data suggest that the RRM domain of U1-70K is not a major contributor to the association between U1 snRNP and pol II in yeast.

### U1-70K RRM makes a minimal contribution to the interaction between purified pol II and U1 snRNP

**Figure 4.**
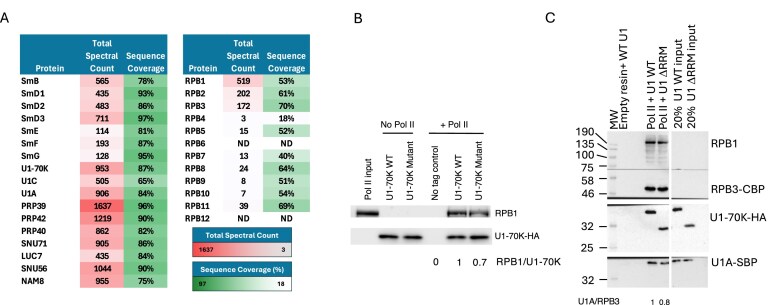
Deletion of the RRM domain in purified U1 snRNP has minimal impact on its interaction with purified pol II. (**A**) Mass spectrometry analysis of the purified U1 snRNP sample shows it contains most of the pol II subunits. ND, not detected. (**B**) U1 snRNP carrying U1-70K WT or quintuple mutant was immobilized on IgG resin and used to pull down purified pol II. The results were analyzed using Western blot, with U1-70K and RPB1 detected by an anti-HA and the anti-RPB1 antibody 8WG16, respectively. RPB1/U1-70K ratio is quantified and normalized to WT U1-70K. Shown here is a representative image from three biological replicates. (**C**) Purified pol II (CBP-tagged) was immobilized on calmodulin resin and used to pull down purified U1 snRNP WT or ΔRRM, which was then analyzed by Western blot. U1-70K and U1A components of the U1 snRNP were detected using an anti-U1-70K antibody and an anti-SBP antibody, respectively. RPB1 and RPB3 were detected by the anti-RPB1 antibody 8WG16 and anti-CBP antibody, respectively. U1A/RPB3 ratio is quantified and normalized to WT U1 snRNP. Shown here is a representative image from two biological replicates.

In the above pulldown experiments from cell lysate, the U1 snRNP and pol II association we observed could be through their direct interactions, but some of this association may also be contributed by both complexes binding to pre-mRNA, which could potentially blunt the difference in direct U1 snRNP and pol II interaction elicited by U1-70K mutants. To rule out this possibility, we evaluated the direct interaction between pol II and U1 snRNP using purified complexes. We purified U1 snRNP using a SBP (Streptavidin Binding Peptide)-TEV-protein A tag on U1A from yeast. Mass spectrometry analysis of the purified U1 snRNP sample shows it contains most of the pol II subunits (Fig. 4A), consistent with the interaction between U1 snRNP and pol II. We next purified pol II using a TAP tag on RPB3 followed by extensive high salt wash (from 200 to 500 to 750 mM NaCl) to remove any potentially tethering DNA or RNA. The purified pol II had an A260/A280 ratio of 0.6, indicating the absence of nucleic acid contamination. We first used purified U1 snRNP WT or quintuple mutant (E122K/K125E/K129E/F130A/ K156E) on IgG resin to pull down purified pol II. We found that the U1 mutant pulls down only slightly less pol II than the WT (Fig. 4B, note that purified U1 snRNP itself does not show a pol II signal in Western blot after extensive washing in the pull down experiment). We next used pol II (containing CBP tag on RPB3) on calmodulin resin to pull down purified U1 snRNP containing either U1-70K WT or ΔRRM (residues 108–188 deleted). We found that pol II pulls down slightly less U1 snRNP ΔRRM compared to the WT (Fig. 4C), although removing the RRM is far from abolishing the interaction, as would be expected based on the human U1 snRNP and pol II cryo-EM structure [7] and our biochemical data (Fig. 1).

### Prp40 makes a substantial contribution to the interaction between U1 snRNP and pol II

Given that U1-70K does not seem to be making major contributions to the interaction between U1 snRNP and pol II (Figs 3 and 4), while U1 snRNP and pol II clearly interact, another U1 snRNP component may be making a more important contribution to this interaction. It has been previously shown that Prp40 interacts with pol II [5]. We therefore evaluated the interaction between Prp40 and pol II and compared it with the interaction between U1-70K and pol II.

**Figure 5.**
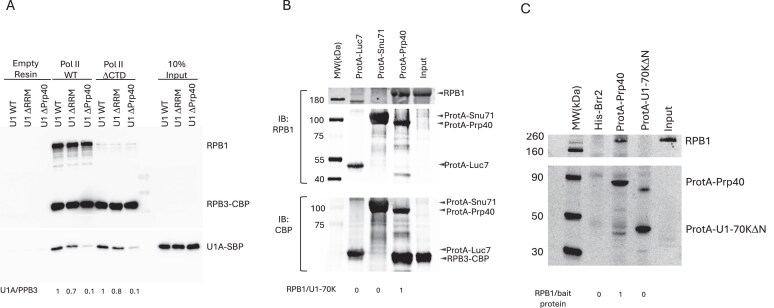
Pol II interacts with Prp40 much more strongly than with U1-70K, and CTD truncation does not affect these interactions. (**A**) Purified pol II WT or ΔCTD (both were CBP tagged) was immobilized on calmodulin resin and used to pull down purified U1 snRNP WT or ΔRRM or ΔPrp40/Luc7/Snu71. Pol II was probed with both the anti-RPB1 antibody 8WG16 (which cannot recognize pol II ΔCTD) and an anti-CBP antibody (which recognizes RPB3-CBP). U1 snRNP was probed with an anti-SBP antibody that recognizes U1A-SBP. U1A/RPB3 ratio is quantified and normalized to WT U1 snRNP. Shown here is a representative image from three biological replicates. (**B**) Purified protein A-tagged Prp40 or Snu71 or Luc7 were immobilized on IgG resin and used to pull down purified pol II, which was probed by the anti-RPB1 antibody 8WG16 or anti-CBP antibody in Western blot. In panels (B) and (C), the primary and secondary IgG antibodies also recognize protein A-tagged Prp40, Snu71, or Luc7 via their protein A tag. IB: immunoblot. U1-RPB1/U1-70K ratio is quantified and normalized to ProtA-Prp40. Shown here is a representative image from three biological replicates. (**C**) Purified protein A-tagged Prp40 or U1-70K ΔN (residues 1–97 deleted) were immobilized on IgG resin and used to pull down purified pol II, which was probed by the anti-RPB1 antibody 8WG16 in Western blot. His-tagged yeast protein Brr2 is an unrelated protein used as a negative control. RPB1/bait protein ratio is quantified and normalized to ProtA-Prp40. Shown here is a representative image from three biological replicates.

To this end, we took advantage of a yeast strain carrying Luc7 with an inducible degron and TAP-tagged U1A (for U1 snRNP purification) that we had previously generated [14]. We have previously shown that Luc7 depletion also removes Prp40 and Snu71 from U1 snRNP [14]. We modified the CBP in the TAP tag with SBP in this strain, so that it does not carry the same CBP tag that is on RPB3 of pol II. We purified U1 snRNP without Prp40, Luc7, and Snu71 from this strain as well as U1 snRNP carrying U1-70K with the RRM domain deleted (Supplementary Fig. S1). We used purified pol II to pull down purified U1 snRNP WT, U1-70K ΔRRM, or ΔPrp40/Luc7/Snu71 and detect the amount of U1 snRNP in the pull down using an anti-SBP antibody. We showed that pol II pulls down slightly less U1 snRNP ΔRRM (Fig. 5A), similar to our observation in Fig. 4C. On the other hand, pol II pulls down dramatically less U1 snRNP ΔPrp40/Luc7/Snu71 compared to the WT (Fig. 5A), indicating that Prp40/Luc7/Snu71 contributes more substantially to the interaction with pol II than U1-70K.

To evaluate if the CTD of pol II is important for its interaction with Prp40/Luc7/Snu71, we inserted a PreScission site between residues 1513 and 1514, which are between the main body of RPB1 and its CTD. After purifying pol II, we cleaved off the CTD using PreScission protease and used the pol II ΔCTD (Supplementary Fig. S1) to pull down various U1 snRNPs. We showed that the pulldown pattern is essentially identical to that of WT pol II (Fig. 5A), indicating that the CTD does not play a major role in the interaction between pol II and Prp40/Luc7/Snu71 (as well as in the weak interaction between U1-70K and pol II). This result is consistent with our observation that using a TAP tag on RPB3 to pull down either WT or CTD-truncated pol II yields similar amounts of associated U1 snRNA (Fig. 2).

To determine whether Prp40 alone in the Prp40/Luc7/Snu71 trimer is sufficient to interact with Pol II, we purified protein A-tagged Prp40 alone on IgG resin from yeast. We showed that it can efficiently pulldown purified pol II which is detected using the anti-RPB1 antibody 8WG16 or an anti-CBP tag against RBP3-CBP (Fig. 5B). On the other hand, protein A-tagged Snu71 or Luc7 alone does not pull down any significant amount of pol II (Fig. 5B).

We further purified protein A-tagged U1-70K ΔN (residues 1–97 deleted but RRM domain remained intact, since we were unable to express full-length U1-70K) from yeast and used it or protein A-tagged Prp40 to pull down purified pol II. We showed that purified Prp40 pulled down significantly more pol II compared to U1-70K ΔN (Fig. 5C), confirming our observation that Prp40 makes a more significant contribution to pol II interaction than U1-70K.

### Multiple domains of Prp40 interact with pol II

**Figure 6.**
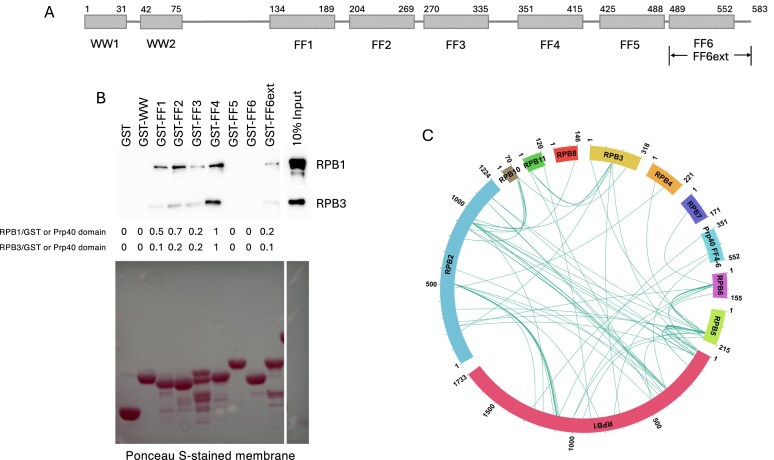
Multiple domains of Prp40 interact with pol II. (**A**) A schematic diagram showing the domain organizations of Prp40. Residue numbers represent the boundary of the constructs used in this experiment. (**B**) Purified GST-fused domains of Prp40 were immobilized on glutathione resin (shown at the bottom is an SDS–PAGE gel stained with ponceau S and note that GST-FF3 has multiple degradation bands) and used to pull down purified pol II, which was probed by the anti-RPB1 antibody 8WG16 or anti-CBP antibody which recognizes RPB3-CBP in Western blot. RPB1/GST or Prp40 domain ratio is quantified and normalized to GST-FF4. Shown here is a representative image from three biological replicates. (**C**) Purified Prp40 FF4-6 was incubated with pol II, crosslinked with DSSO or SDA, and analyzed by mass spectrometry with the identified crosslinks from both crosslinkers displayed in a wheel diagram.

Yeast Prp40 is a 583-residue protein composed of two WW domains followed by six FF domains (Fig. 6A). To decipher which domain of Prp40 interacts with pol II, we expressed and purified GST-fused WW, FF1, 2, 3, 4, 5, 6, and 6 extension domains in *E. coli* and used them to pull down purified pol II. We found that the FF4 domain has the strongest interaction, followed by FF2, FF1, FF3, and the extension downstream of FF6, while the WW, FF5, and FF6 domains have no substantial interactions (Fig. 6B).

**Figure 7.**
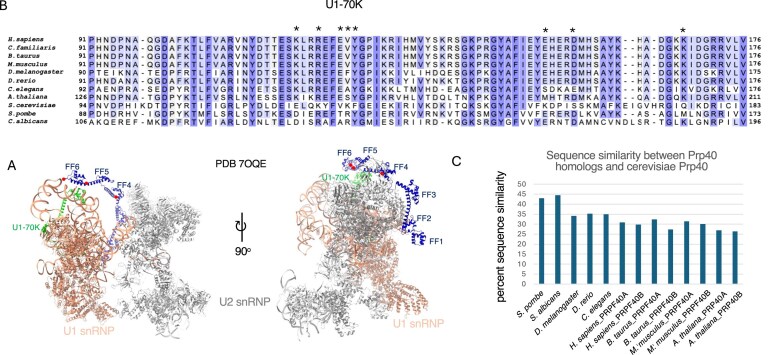
Sequence comparison of U1-70K or Prp40 homologs among different species. (**A**) Ribbon diagram of the yeast pre-A complex showing the position of Prp40 relative to U1-70K and U2 snRNP and a 90-degree rotated view. Red spheres in Prp40 indicate sites identified in crosslinking and mass spectrometry experiments. (**B**) Multiple sequence alignment of the region of U1-70K involved in interaction with pol II among different species. Blue designates conserved residues (with darker colors indicating conservation among more species than the lighter colors). * designates residues important for the interaction with pol II based on the cryo-EM structure of human U1 snRNP and pol II [7] . (**C**) Sequence similarities between Prp40 homologs and *S. cerevisia*e Prp40. Sequence alignment was performed using the EMBL-EBI EMBOSS Needle Pairwise Sequence Alignment tool.

We next incubated purified pol II with the Prp40 FF4-6 domain and treated the sample with the DSSO or SDA crosslinker. Mass spectrometry analysis of the crosslinked sample revealed that the FF4-6 domains were crosslinked to RPB1 (Fig. 6C and Supplementary Table S1), supporting the interaction between the two proteins observed in pull-down analyses (Fig. 6B). Mapping the crosslinking residues (K363, E489, and T511) onto the Prp40 model in the cryo-EM structure of the yeast pre-A complex (Fig. 7A) [3] and the NMR structure of the yeast Prp40 FF6 domain (Supplementary Fig. S2) [20] indicates that these residues are solvent-exposed and are potentially located at or close to the interaction interface with pol II.

## Discussion

U1 snRNP is a major player in the physical coupling between transcription and splicing, connecting the spliceosome to pol II. In this paper, we showed that U1-70K does not play a major role in interacting with pol II in yeast (Figs 2 –4), in contrast to the human system. On the other hand, Prp40 seems to be the main player mediating the interaction between U1 snRNP and pol II (Fig. 5). We found that multiple domains of Prp40 (including FF1-4 and FF6 extension) directly interact with purified pol II in pull-down assays using GST-Prp40 domains purified from *E. coli*, which was consistent with our crosslinking and mass spectrometry analyses (Fig. 6). Our results showed that the Prp40 WW domain does not pull down a substantial amount of pol II, consistent with a previous observation by Gornemann *et al*. that the WW domain is not required for the co-transcriptional recruitment of U1 snRNP and early spliceosomal assembly [21].

Prp40 has been previously reported to interact with pol II by Morris and Greenleaf, although this interaction was thought to be with the CTD of pol II [5]. In Morris and Greenleaf, GST-fused Prp40 purified from *E coli* was run on SDS–PAGE, transferred to nitrocellulose membrane, and incubated with radiolabeled pol II CTD to detect binding in a Far Western assay. The GST-Prp40 proteins used in Far Western analyses were denatured on SDS–PAGE, which may generate artifacts in interaction studies. The GST-Prp40 FL and domains used in these studies also contained a substantial amount of contaminating proteins, potentially complicating the interpretation of the results. In our experiments, using highly purified pol II and U1 snRNP, we showed that deleting the entire CTD from pol II had no effect on the interaction between pol II and U1 snRNP (Fig. 5A). Because Prp40 does not rely on the CTD to interact with Pol II, it is possible that U1 snRNP’s recruitment to Pol II may not depend on the numerous regulatory events occurring at the CTD. This mechanism appears to be conserved in humans, where the interaction between pol II and U1 snRNP is mediated by RPB2 and U1-70K and is also independent of the RPB1 CTD.

Our results revealed interesting differences in the physical coupling between U1 snRNP and pol II in yeast and human. In yeast U1 snRNP, Prp40 is a stably associated component and its FF6 domain interacts with the RRM domain of U1-70K on its pol II-binding surface (Fig. 7A). This interaction likely prevents the RRM domain of U1-70K from interacting with pol II. In humans, PRPF40a and PRPF40b (the human homologs of Prp40) are alternative splicing factors [22–24] that are not integral components of U1 snRNP. The lack of a stable association between human PRPF40s and U1 snRNP, coupled with the exposed RRM domain of U1-70K in the absence of PRPF40s, providing an opportunity for U1-70K to serve as the primary mediator of the interaction between U1 snRNP and pol II. This may also allow PRPF40s to function as alternative splicing factors without compromising the coupling between splicing and transcription. Indeed, multiple sequence alignment (Fig. 7B) shows that most key residues in U1-70K that are important for pol II binding are highly conserved among higher eukaryotes with extensive alternative splicing, but are not conserved in *Saccharomyces cerevisiae, Schizosaccharomyces pombe*, and *Candida albicans*, which have limited or no functional alternative splicing [25–27]. Notably, Montanes and colleagues identified only 262 genes with alternative splicing isoforms out of ∼3010 intron-containing genes (∼8.7%) in *S. pombe* [26]. Thus although alternative splicing in *S. pombe* is more prevalent than previously appreciated, it remains far more limited than higher eukaryotes. Furthermore, while Stepankiw *et al*. identified a large number of alternative splicing events in *S. pombe*, the majority of these represent aberrant splice site usage and there is limited functional alternative splicing [25]. This difference in U1-70K also correlates with the greater sequence similarity between Prp40 from *S. pombe* or *C. albicans* and *S. cerevisiae* compared to Prp40 from higher eukaryotes (Fig. 7C).

We found that yeast pol II also associates more strongly with U2 snRNP than other snRNPs (Fig. 2), although it remains unclear if this interaction is direct. Similar associations were observed by Robert *et al*. who found that a complex containing RNA pol II purified using beads coupled to transcription elongation factor SII also contained splicing factors U2AF^65^, SR proteins, and proteins from multiple snRNPs including U2 snRNP [28]. The molecular details of the U2 snRNP and pol II interaction are not yet clear. TAT-SF1, a human U2 snRNP component (Cus2 in yeast), has been shown to associate with a pol II elongating complex, although it is unclear if it directly interacts with pol II [29]. The cryo-EM structure of the yeast pre-A complex shows that the FF4 domain of Prp40 primarily bridges the interaction between U1 and U2 snRNPs [3], but it remains possible for pol II to interact with other domains and surfaces of Prp40 (Fig. 7A). Thus, U2 snRNP could associate with pol II either directly without contacting U1 snRNP or indirectly through its interaction with U1 snRNP, potentially forming a pol II–U1 snRNP–U2 snRNP complex in either scenario. The recruitment of both U1 and U2 snRNPs by pol II may facilitate the recognition of BPS by U2 snRNP and the formation of the pre-A complex.

The coupling between transcription and splicing is potentially important for efficient co-transcriptional splicing. Interestingly, splicing seems to also influence transcription, potentially through their coupling. For example, Caizzi *et al*. found that inhibition of pre-mRNA branch site recognition by U2 snRNP increases the duration of pol II pausing in the promoter–proximal region, hinders the recruitment of the pause release factor P-TEFb, and reduces pol II elongation velocity at the beginning of genes [30]. Our investigation of the U1 snRNP and pol II interaction in yeast is a step towards better understanding the function of this coupling in the future.

## Supplementary Material

gkag581_Supplemental_Files

## Data Availability

The data underlying this article are available in PRIDE at https://www.ebi.ac.uk/pride/, and can be accessed with Project ID PXD078573. All other data supporting the findings of this study are available within the article and its Supplementary Data files.

## Notes

Present address: College of Plant Protection, Shandong Agricultural University, Tai’an, Shandong 271000, China
