# Supplementary material for: U1 snRNP and RNA polymerase II interaction is predominantly mediated by Prp40 rather than U1-70K in yeast
Source: Nucleic Acids Res. 2026 Jun 10;54(11):gkag581. doi: 10.1093/nar/gkag581 (PMC13250567; doi:10.1093/nar/gkag581)
Supplement: gkag581_Supplemental_Files [file gkag581_supplemental_files.zip › 050626035158_supplementary_figures.pdf]

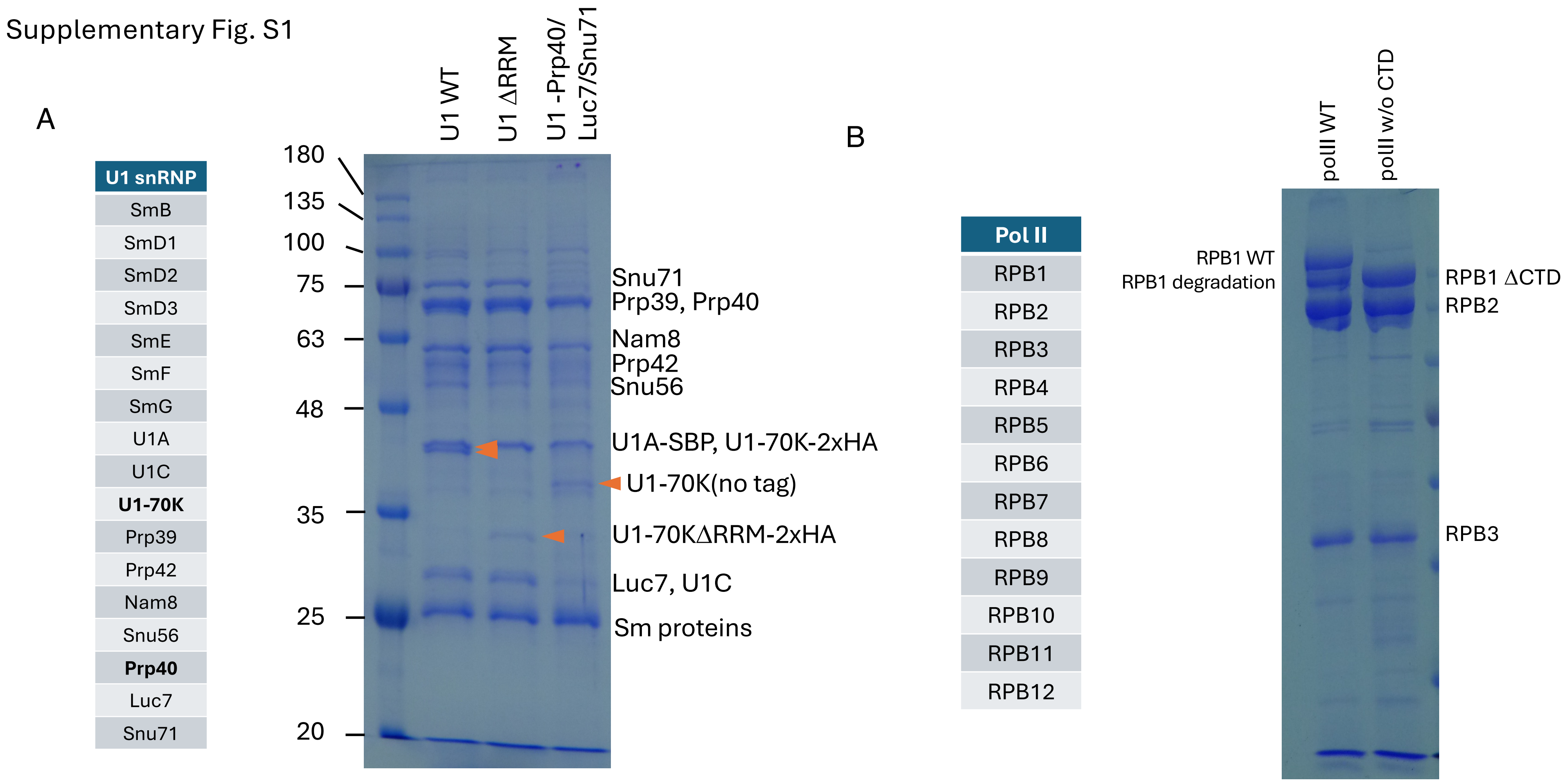

Supplementary Fig. S1. Various purified U1 snRNP and pol II complexes.

A. WT, ΔRRM, and Prp40/Luc7/Snu71 depletion shown on SDS PAGE with Coomassie stain. The protein components of yeast U1 snRNP are also shown with the two proteins most relevant to this study highlighted in bold.

B. pol II WT and CTD truncation shown on SDS PAGE with Coomassie stain. The protein components of yeast pol II are also shown.

Supplementary Fig. S2

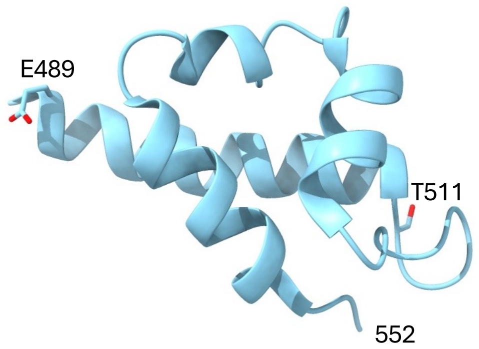

Supplementary Fig. S2. Residues identified from the crosslinking and mass spectrometry experiments are mapped on the NMR structure of yeast Prp40 FF6 domain (PDB 2FKD).
